# Supplementary material for: Sensitive Detection of Industrial Pollutants Using Modified Electrochemical Platforms
Source: Nanomaterials (Basel). 2022 May 23;12(10):1779. doi: 10.3390/nano12101779 (PMC9142962; doi:10.3390/nano12101779)
Supplement: Supplementary file 1 [file nanomaterials-12-01779-s001.zip › nanomaterials-1734534-supplementary.pdf]

Supplementary materials

Sensitive Detection of Industrial Pollutants Using Modified Electrochemical Platforms

Alessio Di Tinno[1], Rocco Cancelliere[1], Pietro Mantegazza[1], Antonino Cataldo[2,3], Alesia Paddubskaya[4], Luigi Ferrigno[5], Polina Kuzhir[6], Sergey Maksimenko[4], Mikhail Shuba[4], Antonio Maffucci[3,5], Stefano Bellucci[2], and Laura Micheli[1,*]

[1] Department. of Chemical Science and Technologies, University of Rome "Tor Vergata", 00133 Rome, Italy;
alessio.ditinno@gmail.com (A.D.T.); roccocancelliere@gmail.com (R.C.);
mantegazza.pietro@gmail.com (P.M.)
[2] DISPREV Laboratory, Casaccia Research Center, ENEA, Rome, Italy; antonino.cataldo@enea.it (A.C.)
[3] National Institute of Nuclear Physics, Frascati National Laboratories, 00044 Frascati (RM), Italy; stefano.bellucci@lnf.infn.it (S.B.)
[4] Institute for Nuclear Problems, Belarusian State University, 220007, Minsk, Belarus;
paddubskaya@gmail.com (A.P.); sergey.maksimenko@gmail.com (S.M.);
mikhail.shuba@gmail.com (M.S.)
[5] Department of Electrical and Information Engineering, University of Cassino and Southern Lazio,
03043 Cassino, Italy; ferrigno@unicas.it (L.F.), maffucci@unicas.it (A.M.)
[6] Department of Physics and Mathematics, Institute of Photonics, University of Eastern Finland, 80200, Joensuu, Finland; polina.kuzhir@uef.fi (P.K.)
* Correspondence: laura.micheli@uniroma2.it
† These authors contributed equally to this work.

S1 - FT-IR characterization of Graphene Nanoplatelets

The FT-IR characterization of the GNP produced for this work is provided in Fig.S1. This characterization is usually used to observe the fuctionalization on GNP due to the fact that GNP does not have distinctive peaks due to the IR selection rules. The analysis confirmed the Raman results: the MW irradiation did not oxidized the GNP. The spectrum, acquired after 30 s of irradiation (that exceed the time of expansion process, about 10 s) does not show peak nor in the region of carbonyl and carboxyl groups (about 1600cm^{-1}) neither in the region of hydroxyl group (about 3000cm^{-1}). The absent of functional groups and consequently low intensity of signals highlight the background and in particular the signals of sample holder, the cell in diamond used for the ATR technique.

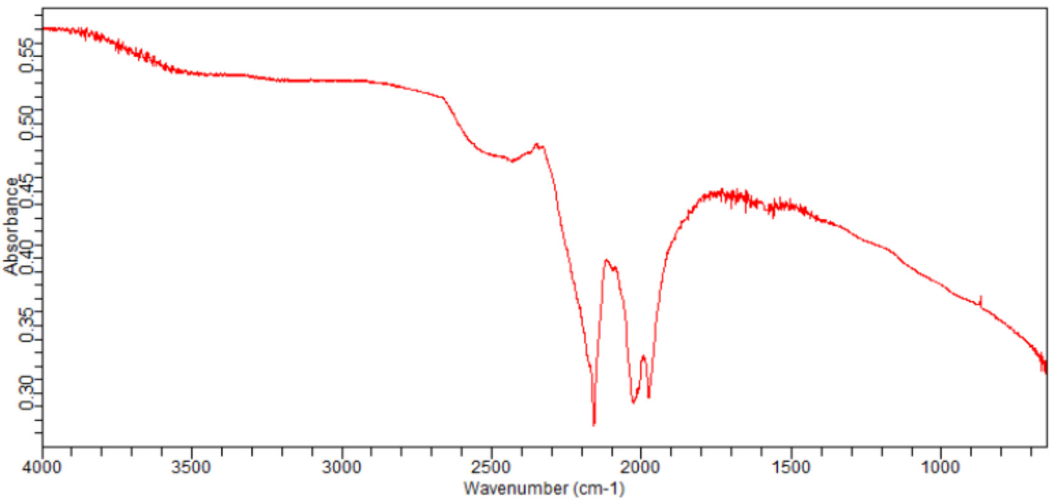

Figure S1. Infrared spectrum of GNP after MW irradiation. As it is possible to observe, the main feature of oxidation, such as carbonyl, hydroxyl and carboxylic groups, are absent. In the region 2600-1900 cm-1, the peaks are ascribed to the sample holder, a diamond cell. The spectrum (128 scans, resolution: 2 cm^{-1}) was acquired with the attenuated total reflection technique using a Cary 630 spectrometer (Agilent) endowed with a diamond cell.

S2. Dose-response voltammograms

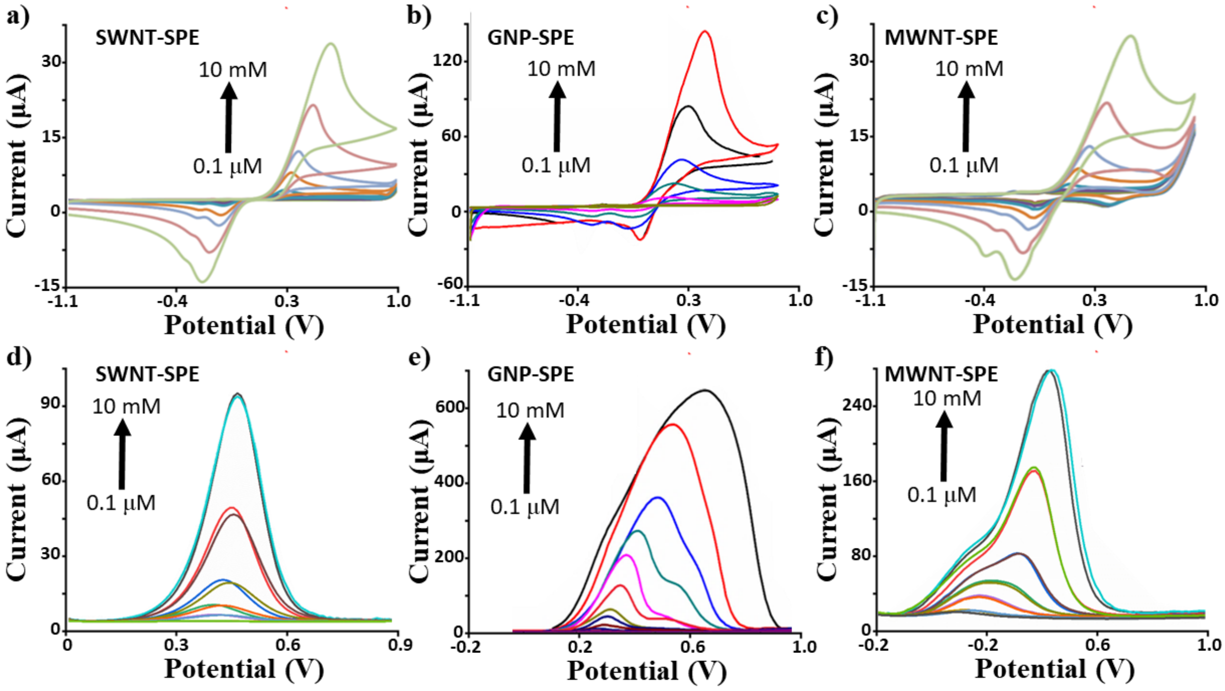

Figure S2. Dose-response voltammograms. (**a–f**) Comparison of the CVs and SWVs for different platforms (SWNT, MWNT, GNP and bare SPE) at several HQ concentrations (from 0.1 to 1µM) in water. Curves of one representative CNMs-modified SPE sensor of at least 6 analyzed platforms are presented.
